# Functionally and morphologically damaged mitochondria observed in auditory cells under senescence-inducing stress

**DOI:** 10.1038/s41514-017-0002-2

**Published:** 2017-01-25

**Authors:** Teru Kamogashira, Ken Hayashi, Chisato Fujimoto, Shinichi Iwasaki, Tatsuya Yamasoba

**Affiliations:** 1 Department of Otolaryngology and Head and Neck Surgery, University of Tokyo, 7-3-1, Hongo, Bunkyo-ku, Tokyo, 113-8655 Japan; 2 Department of Otolaryngology, Kamio Memorial Hospital, Tokyo, 101-0063 Japan; 3 Department of Otolaryngology, School of Medicine, Keio University, Tokyo, 160-8582 Japan

## Abstract

We aimed at determining the mitochondrial function in premature senescence model of auditory cells. Short exposure to H_2_O_2_ (1 h, 0.1 mM) induced premature cellular senescence in House Ear Institute-Organ of Corti 1 auditory cells. The transmission electron microscopy analysis revealed that damaged mitochondria and autophagosomes containing dense organelles appeared in the auditory cells after short exposure to H_2_O_2_. The branch and junction parameters of the skeletonized image of the mitochondria were found to decrease significantly in H_2_O_2_-treated cells. A branched reticulum of tubules was poorly formed, featuring coexistence of numerous tiny clusters along with few relatively large entities in the H_2_O_2_-treated cells. In terms of bioenergetics, H_2_O_2_-treatment led to the dose-dependent decrease in mitochondrial membrane potential in the auditory cells. The fragmented mitochondria (fusion < fission) were in a low potential. In addition, the potential of hyperfused mitochondria (fusion > fission) was slightly lower than the control cells. The short-time exposure of live auditory cells to H_2_O_2_ damaged the mitochondrial respiratory capacity without any effect on the baseline ATP production rates. The vulnerability of the mitochondrial membrane potential to the uncoupling reagent was increased after H_2_O_2_ treatment. Our findings indicated that the mitochondrial dysfunction due to the decline in the O_2_ consumption rate should be the first event of premature senescence process in the auditory cells, resulting in the imbalance of mitochondrial fusion/fission and the collapse of the mitochondrial network.

## Auditory system senescence: Mitochondrial dynamics and respiration

The mitochondrial morphology and physiology could influence the process of age-related hearing loss. Prof. Tatsuya Yamasoba’s research group at the University of Tokyo has examined the functional changes of mitochondria in terms of its respiratory function, membrane potential and morphology under premature senescence induced by oxidative stress in an auditory cell line. The morphological and functional mitochondrial damage were observed as the respiratory capacity deficiency and the fluctuation of the fusion/fission balance. Their results provide evidence of the fundamental interdependence between mitochondrial metabolic activity and its network structure in premature senescence process of auditory cells. This is a pioneer study to indicate the influence of mitochondrial dynamics and respiratory system on the premature senescence process of auditory cells. Further studies into inter cellular communication including cytoskeleton and nucleus can help us understand the etiology underlying age-related hearing loss.

## Introduction

Age-related hearing loss (ARHL), known as presbycusis, is one of the serious problems in the super-aging society.^1–3^ The latest finding indicated that hearing loss was independently associated with accelerated cognitive decline and incident cognitive impairment in community-dwelling older adults.^4^ ARHL is characterized by an age-dependent decline of auditory function attributable to the loss and dysfunction of hair cells, spiral ganglion cells, and stria vascularis cells in cochlear of the inner ear.^5^ It is also characterized by the noise-induced neurodegeneration.^6^ However, the molecular mechanism of ARHL is still unclear.

Mitochondria regulate a number of cellular processes including cellular metabolism, senescence, and death. Therefore, the maintenance of mitochondrial homeostasis plays a crucial role in cellular fate decisions. A recent study demonstrated that mitochondrial dysfunction was among the nine tentative hallmarks that represent common denominators of aging in different organisms, with special emphasis on mammalian aging.^7^ The mitochondrial theory of aging is based on the premise that cumulative damage caused by the production of free radicals can alter the mitochondrial DNA.^8,9^ Indeed, a recent study indicated that the mitochondrial redox imbalance and mutation in mitochondrial DNA might be collaboratively involved in the process of cochlear senescence in the aging stress.^5,10^ Many other reports have also described the relationship between oxidative stress and mitochondrial dysfunction in ARHL.^11^ However, the influence of mitochondrial morphology and physiology on ARHL is still unclear. Mitochondrial morphology is very dynamic in nature and can shift between fragmented structures and filamentous network, via mitochondrial fusion and fission events.^12^ Mitochondrial dynamics and spatial localization are linked to mitochondrial and cellular functions.^13–15^ Impairment of the regulation and function of mitochondria could severely affect cellular homeostasis and result in aging and several diseases including metabolic disorder, cancer, and neurodegeneration.^16^ An important point in this issue is referred as to the implication of the disturbance of the mitochondrial fusion and fission processes, which routinely regulates the mitochondrial network homeostasis in the process of cell aging.^17,18^ However, there has been no report on the influence of mitochondrial dynamics on ARHL.

In terms of bioenergetics, the mitochondrial dysfunction in aged mammals exhibits a diminished capacity of adenosine triphosphate (ATP) production, decreased membrane potential, as well as decreased mitochondrial respiratory chain enzyme activities.^19–21^ Auditory cells, including cochlear hair cell, are also highly dependent on the energy provided by mitochondrial ATP production and respiration.^22^ However, the relationship between aging and the bioenergetics of mitochondria in auditory cells remains unclear.

On the basis of these interesting in vitro and in vivo findings, we decided to investigate the role of mitochondrial network integrity on auditory bioenergetics and function in ARHL. Then, conditionally immortalized mouse auditory cells, House Ear Institute-Organ of Corti 1 (HEI-OC1) auditory cells,^23^ were incubated with a short time exposure to H_2_O_2_, which induced a senescent phenotype.^24^ Here, we examined the mitochondrial metabolic activity and its network structure under senescence-inducing stress of the auditory cells.

## Results

### Short exposure to H_2_O_2_ induced premature cellular senescence in HEI-OC1 cells

**Fig. 1:**
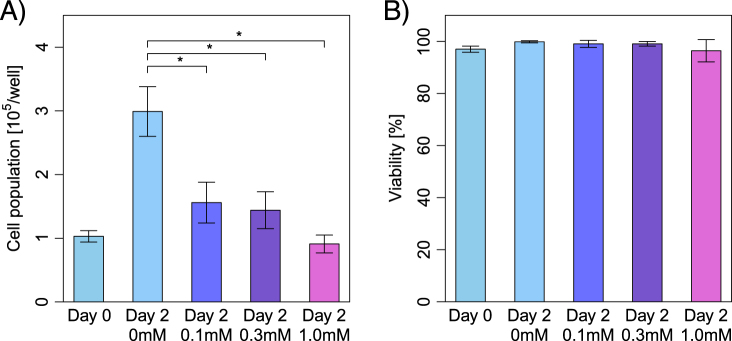
Short exposure to H_2_O_2_ induces a decrease of population doubling in a dose-dependent manner, but does not affect the cell viability in HEI-OC1 cells. **a** Population doubling time. Population doubling experiments were performed in duplicate. **b** Cell viability. Cell viability was determined by trypan blue staining at the indicated times after short exposure (1 h) with H_2_O_2_ (0 μM, 100 μM, 300 μM, and 1 mM). All values are showed as mean ± SD from three or more independent studies, *n* = 6 per group. *P < 0.05

Premature cellular senescence can be induced by exposing H_2_O_2_ in a concentration-dependent manner.^25,24^ First, we evaluated the population doubling rates and viability of HEI-OC1 cells under several concentrations of H_2_O_2_ exposure in order to determine whether oxidative stress can induce the premature senescence of auditory cells. The HEI-OC1 cells were treated with different concentrations of H_2_O_2_ for 1 h, replaced with normal medium, and incubated under permissive conditions for two days. The population doubling rate significantly decreased after 2 days with short exposure (1 h) of H_2_O_2_ in a dose-dependent manner (Fig. 1a) while the exposure with 1 mM H_2_O_2_ for 1 h had no effect on the cell viability ratio (Fig. 1b). These results suggest that the above-mentioned conditions (exposure with 100 μM, 300 μM and 1 mM H_2_O_2_ for 1 h, and incubation under permissive conditions for 2 days) could be used as a premature cellular senescence model of auditory cells from the point of the cellular-function.

### Short exposure to H_2_O_2_ induced morphological and ultrastructural changes of mitochondria in HEI-OC1 cells

**Fig. 2:**
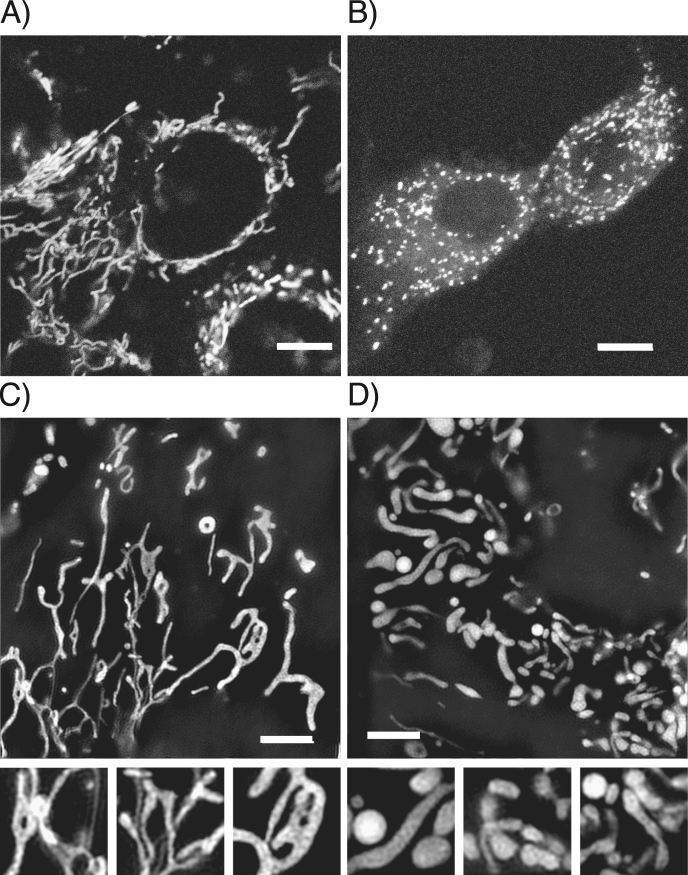
Change in mitochondrial morphology in H_2_O_2_-exposed cells. Short exposure to H_2_O_2_ induces fine structural changes of mitochondria in HEI-OC1 cells. Cells were dyed with TMRE MMP fluorescence. **a** Control cells captured with confocal microscopy. The form of the mitochondria is string shaped (Scale bar, 10 μm). **b** H_2_O_2_-exposed cells. The shape of the mitochondria is segmented in small balloon shape (Scale bar, 10 μm). **c** Control cells captured with N-SIM (Scale bar, 5 μm, Small box, 2 times magnified image). **d** Cells exposed to H_2_O_2_ and captured with N-SIM. The thickness of the mitochondria is larger than the control cells (Scale bar, 5 μm, Small box, 2 times magnified image)

As shown in Fig. 2, early alterations in the shape of mitochondria of auditory cells at day 1 after a short exposure to H_2_O_2_ (1 mM, 1 h) were observed in the confocal microscope and the Nikon’s structured illumination microscope (N-SIM). The control group cells had dynamic mitochondria with a good balance of fusion and fission (Fig. 2a, c) while the fragmented mitochondria with the balloon-like shape or the hyperfused mitochondria with enlarged shape were observed in H_2_O_2_-exposed cells (Fig. 2b, d).

**Fig. 3:**
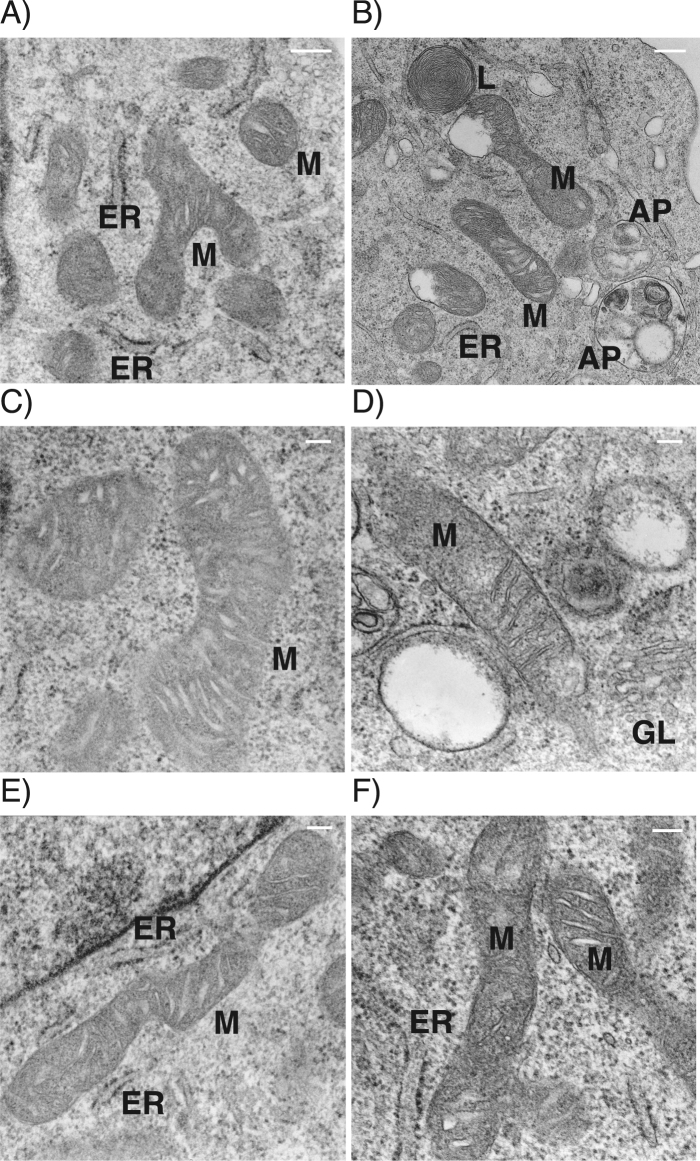
Change in mitochondrial ultrastructures in H_2_O_2_-exposed cells. Typical TEM images of HEI-OC1 cells. Cells were treated as in confocal microscopy experiments. **a**, **c**, **e** Control cells. **b**, **d**, **f** Cells at one day after treatment with 1 mM H_2_O_2_ for 1 h. *AP* autophagosome, *ER* endoplasmic reticulum, *GL* goldi body, *L* lysosome, *M* mitochondria. Scale bar, **a**, **b** 200 nm, **c**, **d**, **e**, **f** 100 nm

We also examined ultrastructural changes of the mitochondria after short-exposure to H_2_O_2_ (1 mM, 1 h) using transmission electron microscopy (TEM). The control group cells exhibited healthy normal appearing mitochondria and endoplasmic reticulum (ER) (Fig. 3a, c, e). In contrast, H_2_O_2_-exposed group cells (1 mM, 1 h) had accumulated the damaged mitochondria and autophagosomes containing lucent materials and dense organelles one day after the treatment (Fig. 3b, d). Autophagosomes appeared close to the damaged mitochondria and ER, having round and double membrane structures (Fig. 3b). The enlarged mitochondria showing hyperfusion or the fragmented mitochondria showing fission were intermingled in the cells (Fig. 3f).

### Short exposure to H_2_O_2_ resulted in poor mitochondrial network in HEI-OC1 cells

**Fig. 4:**
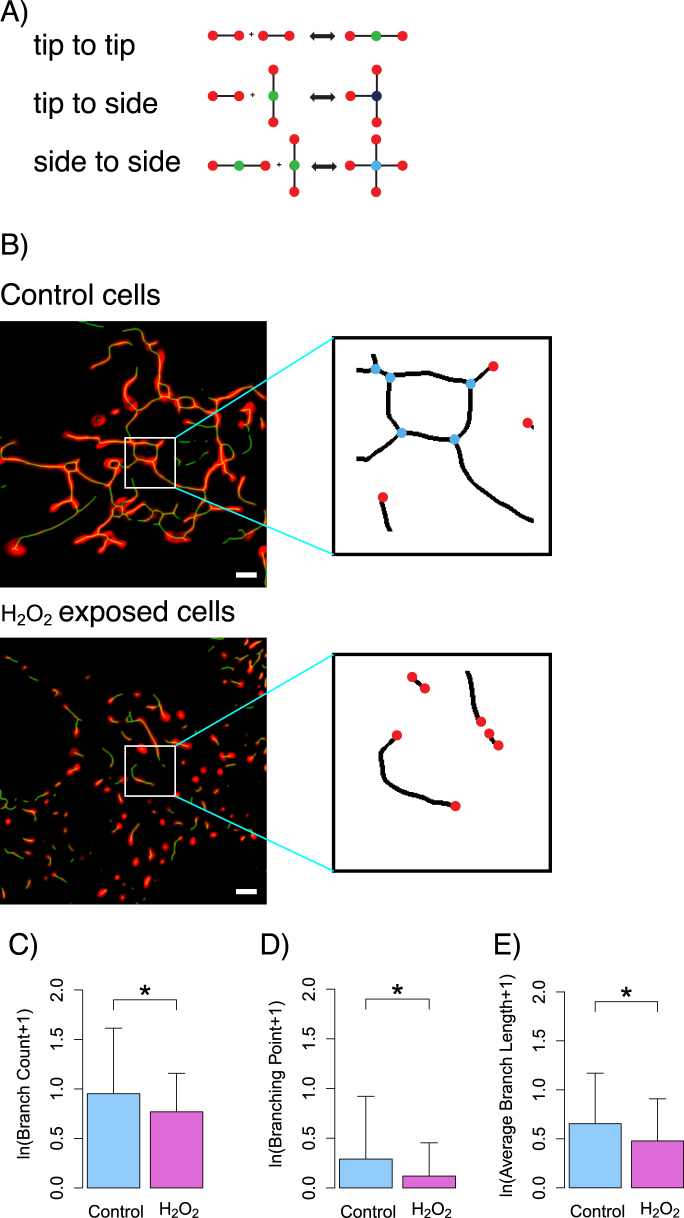
Numerical image analysis of mitochondria changes in H_2_O_2_-exposed cells. Fine structural changes of mitochondria in HEI-OC1 cells were numerically analyzed. Cells were dyed with MitoTracker Orange mitochondrial fluorescence and captured with confocal microscopy. **b** Different types of fusion and fission mechanisms involving process-specific kinds of network nodes (tip-to-tip, tip-to-side or side-to-side). Graph representation of the mitochondrial reticulum using four node types: 1 (*magenta*), 2 (*green*), 3 (*dark blue*), and 4 (*blue*). The reticulum can be represented as a set of linear segments consisting of one or more edges (*black rods*) liking the nodes. **b** Control cells (*upper panel*) and H_2_O_2_-exposed cells (*lower panel*) with bandpass filter and Skeletonize plugin on ImageJ. Skeleton (*left panel*) shows that the red images are band-pass filtered image, and the green lines show the filtered and skeletonized image. Analysis (*right panel*) shows the connection between tip and side. The natural logarithm ln(x) of raw parameter + 1 was used because the parameter’s distribution was closed to log-normal distribution. The natural logarithm of branch count **c**, branching point **d** and average branch length **e** showed a significant decrease in the H_2_O_2_-exposed cells compared to the control cells (*P* < 0.05). Descriptor variables are shown in Supplementary Table 1

The morphological and dynamic changes in mitochondria leave a distinctive footprint on the resulting network structure. The process of fusion and fission can be deduced from the morphological still image analysis. As shown in Fig. 4a, there are different types of fusion and fission mechanisms involving process-specific kinds of network nodes (tip-to-tip, tip-to-side or side-to-side). The fusion and fission dynamics lead to a branched reticulum of tubules. The whole network consists of disconnected clusters of tubules. In order to allow exact quantitative comparison of mitochondrial network structure in the image, we described descriptors with correspondent meanings in Supplementary Table 1. The dynamic mitochondrial network in the control cells consists of tubules with appropriate length and size (Fig. 4b upper panel). On the contrary, there was a coexistence of numerous tiny clusters along with a few relatively large entities in H_2_O_2_-exposed cells, without forming any mitochondrial network (Fig. 4b lower panel). There were significant differences for branch count (Fig. 4c), branching point (Fig. 4d), and average branch length (Fig. 4e) between control cells and H_2_O_2_-exposed cells (*P* < 0.05).

### Brief exposure to H_2_O_2_ decreased mitochondrial membrane potential (MMP) in HEI-OC1 cells

**Fig. 5:**
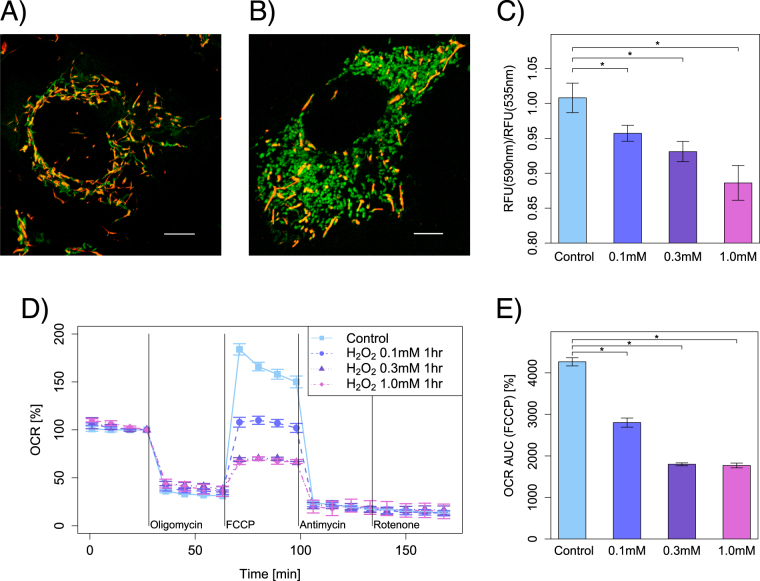
Functionality change in mitochondria with exposure to H_2_O_2_ and the decrease of O_2_ consumption rate in HEI-OC1 cells. Cells with high MMP promote the formation of dye aggregates and fluoresce red; cells with low potential contain monomeric JC-1 and fluoresce green. **a** The mitochondria stained with JC-1 in control cells, and **b** the mitochondrial potential collapse stained with green was induced in H_2_O_2_-exposed cells (1 mM for 1 h). **c** The MMPs were determined by plate reader fluorescent analysis with JC-1 mitochondrial membrane fluorescent dye 1 day after short exposure (1 h) with H_2_O_2_ (0 μM, 100 μM, 300 μM, and 1 mM). Indeed, the ratio of red signal (Relative fluorescence units (RFU) at 590 nm; mitochondrial polarized cells) to green signal (RFU at 535 nm, all cells) was measured. All values are showed as mean ± SD from three or more independent studies, *n* = 6 per group. *P < 0.05. (Scale bar, 10 μm). The O_2_ consumption rates were calculated in relative value from the baseline of O_2_ consumption rate one day after short exposure with H_2_O_2_ (0 μM, 100 μM, 300 μM, 1 mM for 1 h). The mitochondrial complex inhibitors and mitochondrial inner membrane uncoupler were injected sequentially during the measurement, and other conditions were identical to Fig. 1 measurement. The relative value of O_2_ consumption rate on FCCP injection was dose dependently decreased in the H_2_O_2_-exposed cells (0.1 mM: 108%, 0.3 mM: 69%, 1 mM: 66%), although the mean rate was 184% in control cells. All values are showed as mean  ±  SD from three or more independent studies, *n* = 5 per group

The mitochondrial depolarization was also observed in H_2_O_2_-exposed HEI-OC1 cells by using 5, 5’, 6, 6’-tetrachloro-1, 1’, 3, 3’-tetraethyl benzimidazolyl carbocyanine iodide (JC-1) mitochondrial membrane fluorescent dye (Figs. 5a, b). The green emission part showed the state of the membrane with low potential while the red emission part showed the high potential state in JC-1 MMP fluorescence. The dynamic mitochondria in control cells were stained deep orange color (Fig. 5a). The fragmented mitochondria in H_2_O_2_-exposed cells were stained green, and the enlarged hyperfused mitochondria were stained light orange (Fig. 5b).

The red/green ratio showing the status of mitochondrial membrane was significantly decreased in a dose-dependent manner in the auditory cells treated with a short exposure (1 h) to H_2_O_2_ (Fig. 5c). However, the polarized mitochondria did not completely vanish in H_2_O_2_-exposed cells.

### Brief exposure to H_2_O_2_ decreased O_2_ consumption rate of mitochondria but had no effect on ATP production in HEI-OC1 cells

We evaluated the mitochondrial function by calculating the maximum respiratory capacity on the injection of the mitochondrial inner membrane uncoupler, carbonyl cyanide p-trifluoromethoxyphenylhydrazone (FCCP), and the respiratory rate on the injection of oligomycin. The short exposure to H_2_O_2_ resulted in a significant decrease in O_2_ consumption rate in a dose-dependent manner (Figs. 5d, e). The O_2_ consumption rate decreased in response to mitochondrial complex inhibitors or FCCP in the H_2_O_2_-exposed cells as well as in the control cells. The ratio of the respiratory changes following the injection of FCCP showed dose-dependent decreases in contrast to the control group. However, there was no significant difference between them in the other respiratory change rates on the injection of mitochondrial complex inhibitors. The ATP-linked O_2_ consumption rates were also not different between the groups. Beside, the continuous exposure to H_2_O_2_ led to cell death, which showed different types of mitochondrial dysfunction profile. The baseline O_2_ consumption rate decreased in the higher concentrations of the H_2_O_2_ group (300 μM and 1 mM), and the ATP-linked O_2_ consumption rate and the maximum respiratory ratio also decreased (data not shown).

### Brief exposure to H_2_O_2_ induces vulnerability of the MMP that could not be protected with mitochondrial permeability transition pore (mPTP) inhibitor

**Fig. 6:**
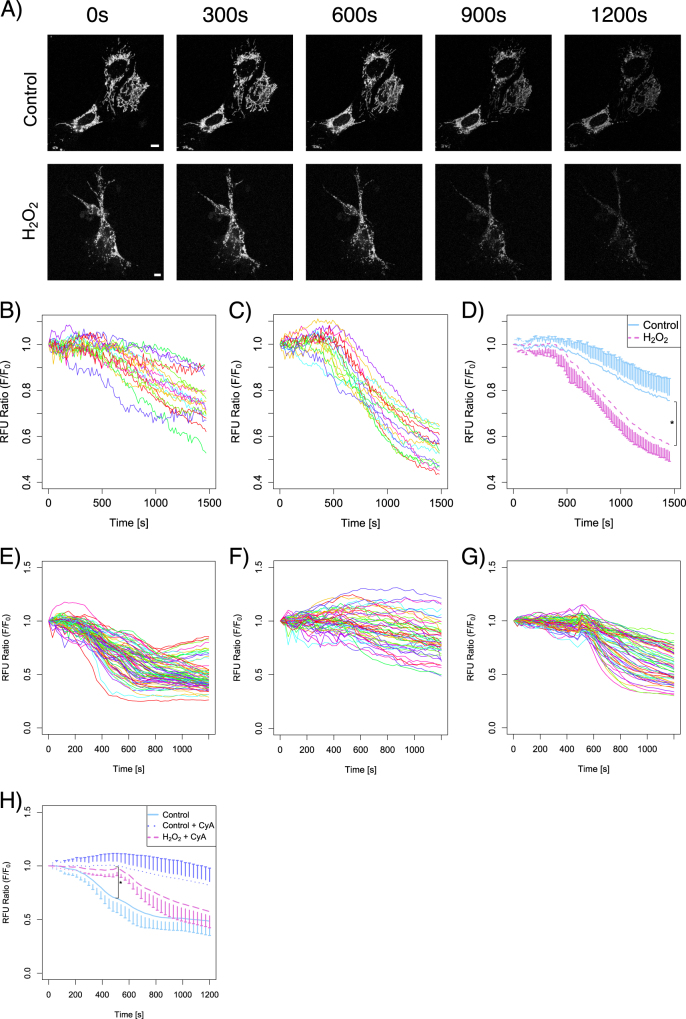
Short exposure with H_2_O_2_ induces vulnerability of the MMP and the mPTP opening. Cells were dyed with 200 nM TMRE MMP fluorescence and the mitochondrial transition pore opening was initiated by the injection of FCCP in the cell culture medium. The fluorescence of TMRE was continuously captured with confocal microscopy. The 1.375 μM FCCP was injected continuously during the TMRE fluorescence recording. The typical time course of the fluorescence cell images showed a continuous decrease of the fluorescence intensity (**a**). Each cell was selected as a ROI and each of the average fluorescence intensities of the ROI was plotted **b**: control group, **c**: H_2_O_2_-exposed group, both from four assays). The average fluorescence intensity decreased quickly in the H_2_O_2_-exposed group (**D)**. The decrease of the relative fluorescent unit showed a significant decrease in the H_2_O_2_-exposed group. *P < 0.05. CyA treatment delayed the decrease of the fluorescence intensities by the injection of FCCP **e**, control group, **f**, CyA 1 μM 20 min treated group, both from four assays). However, the fluorescence intensities decreased in the H_2_O_2_-exposed group **g**, from four assays). The decrease of the average fluorescence intensity showed significant difference between groups (**h**). In the CyA-treated groups, the collapsed times of the MMP were 600 s in both groups. *P < 0.05

We evaluated the functional changes of mPTP. In general, the MMP decreases with the opening of mPTP, which can be triggered by FCCP injection (Fig. 6a). As shown in Figs. 6b–d, in H_2_O_2_-treated cells, the MMP significantly decreased on the administration of FCCP. In the case of the cells treated with mPTP inhibitor, cyclosporine A (CyA), before the FCCP trigger, the MMP deterioration was protected, although the protective effect was partial and the collapse time of the MMP did not decrease (Fig. 6e–h). The O_2_ consumption rate in the CyA treatment showed no difference in baseline and maximum respiratory ratios in the control group. Also, the CyA treatment did not protect the maximum respiratory capacity decrease in the H_2_O_2_-exposed group (Suppl. Fig. 4).

## Discussion

In this study, we demonstrated that the premature senescence induced by oxidative stress was attributable to the impairment of mitochondrial function in the maximal respiration with decreased MMP. In the morphology site, we indicated that the imbalance of mitochondrial fusion and fission plays a crucial role in the aging process of the auditory cells. Our results provide evidence of the fundamental interdependence between mitochondrial metabolic activity and its network structure in premature senescence process of auditory cells (Suppl. Fig. 5) .

We used the short exposure to H_2_O_2_ for 1 h at varied concentrations as a stress inducer in our premature senescence cell model of the auditory cell. H_2_O_2_ has been widely used to achieve oxidative stress-induced premature senescence within a short period of time.^26,27^ In our study, the condition of the cell-treatment targeting the mitochondrial function of auditory cells was decided based on the previous report.^24^ Indeed, short H_2_O_2_ exposure led to the significant decrease in cell population, but had no significant effect on cell viability, suggesting the induction of premature senescence of auditory cells (Fig. 1).

The TEM analysis revealed that damaged mitochondria and autophagosomes containing dense organelles appeared in H_2_O_2_-exposed cells, while mitochondrial fusion and fission were observed in both control and H_2_O_2_-exposed cells (Fig. 3), however, the N-SIM analysis showed that the elongated mitochondria characteristically existed in control cells, while the balloon shaped mitochondria or the remarkably short length of mitochondria appeared frequently in H_2_O_2_-exposed cells (Fig. 2). These results suggest that fragmented mitochondria may have increased due to an imbalance of mitochondrial dynamics (fusion < fission) in the process of auditory premature senescence. Mitochondria are recycled in a dynamic equilibrium between opposing processes of fusion and fission. Fusion produces elongated interconnected mitochondria that form networks, which are involved in facilitating the membrane potential across individual cells.^28^ Fission is needed to control cell quality by replacing damaged mitochondria with new mitochondria, and also facilitates apoptosis during high levels of cellular stress. That means balanced fusion and fission are needed to maintain functionality of mitochondria when cells are affected by a variety of stresses.

In addition, it is very interesting to note that the branch and junction parameters of the skeletonized image of the mitochondria significantly decreased in H_2_O_2_-exposed cells (Fig. 4). Basically, the details of fusion and fission procedures can be deduced from the morphological still image analysis because the underlying dynamic behavior of mitochondria leaves a distinctive and stable footprint on the resulting network structure. Fusion and fission dynamics should lead to the formation of branched reticulum of tubules whose lengths are well approximated by a genomic law and whose mean size in equilibrium is determined by relative rates of these processes.^29^ These results indicate that an imbalance of mitochondrial dynamics and/or inefficient mitochondrial degradation due to dysfunction of autophagy seems to be particularly a crucial factor in premature senescence of auditory cells. Since mitochondrial dysfunction is influenced by the inactivation of the mitochondrial fusion machinery, this consequently leads to smaller mitochondrial entities,^30^ and large numbers of small clusters present in the mitochondrial network are attributable to the formation of autophagosomes.^31^ In terms of bioenergetics, H_2_O_2_ exposure led to the dose-dependent decrease of MMP in the case of auditory cells (Fig. 5c). The fragmented mitochondria (fusion < fission) were in a low potential although the potential of hyperfused mitochondria (fusion > fission) was slightly lower than the control cells (Fig. 5). On the contrary, dynamic mitochondria (fusion = fission) in control cells were in a high potential. These results indicate that premature senescence-related bioenergetic alterations in mitochondrial fusion and fission dynamics play a causative role in mitochondrial dysfunction of auditory cells, supporting the idea that disruption of mitochondrial fusion results in mitochondrial dysfunction.^32,33^ Taken together, the fundamental interdependence between mitochondrial metabolic activity and its network structure exists in auditory cells.^34–36^

Next, we measured the maximum respiratory capacity with XF24 analyzer in order to evaluate the mitochondrial function of live cells in auditory cellular senescence process (Fig. 5d). In the XF24 assay, the maximum respiratory ratio is defined as the increase in the ratio of the O_2_ consumption rate between the basal respiration and the respiration on the injection of the mitochondrial membrane uncoupler, FCCP. The spare respiratory capacity of mitochondria has emerged as an important factor in response to oxidative stress and energetic insufficiency in the recent years.^37–39^ The maximum respiratory capacity is considered as a reserve capacity to protect cells from various stress including energy insufficiency, reactive oxygen species stress and substrate insufficiency.^40^ The maximum respiratory ratio mainly depends on the function of the substrates supplementation.^40^

In this study, the short time H_2_O_2_ exposure was found to damage the mitochondrial respiratory capacity but not the baseline ATP production rates in live auditory cells (Fig. 5d). This functional decline of mitochondrial respiratory system would be the first event in an auditory cellular senescence process. In terms of mitochondrial dynamics, mitochondrial fusion is very important for the maintenance of respiratory capacity in auditory cells having a high metabolic activity.

The vulnerability of the MMP to the uncoupling reagent was seen to increase by the H_2_O_2_ exposure. The FCCP injection triggered the mPTP opening, leading to a decrease in the MMP. The mPTP inhibitor, CyA, protected the membrane potential decrease, but the collapse time of the H_2_O_2_-exposed group did not differ from that of the control group (Fig. 6). The resistance or fragility to the mitochondrial uncoupling have been widely reported in cancer cell models,^41^ cardiomyocyte cell models with cardioprotective drugs or anesthetic agents,^42–45^ and HeLa cell models.^46^ The decrease in the MMP mainly relies on the mPTP function, which can be analyzed with mPTP inhibitor, including CyA or cyclosporin H. However, other factors also affect the MMP decrease, which makes it difficult to interpret the membrane potential changes. The promotion of mPTP with cyclophilin-D in neuronal cell model showed opposite effects on apoptosis and necrosis.^47^ In the current study, the H_2_O_2_ treatment did not affect the collapse time of the membrane potential that decreased with an injection under CyA treatment, but the decrease of the membrane potential after collapse showed a difference, which implicates the factors other than mPTP that could protect membrane potential.

Our study demonstrated that premature senescence could be induced with the functional decline of the mitochondrial respiratory system in auditory cells, and that the decrease in the ATP production-linked respiration was not observed under senescence-inducing stress of the auditory cells. Hence, the mitochondrial dysfunction due to the decline of the O_2_ consumption rate resulted in the imbalance of mitochondrial fusion/fission and the collapse of the mitochondrial network (Suppl. Fig. 5). It has been stated that senescent cells that acquire phenotypic changes may contribute to aging and certain age-related diseases.^48,49^ To our knowledge, this is the first report to indicate the influence of mitochondrial dynamics and mitochondrial respiratory system on the premature senescence process of auditory cells. However, further studies need to be conducted to determine how premature senescence contributes to the etiology underlying ARHL.

## Materials and methods

### Cell culture and culture conditions

HEI-OC1 auditory cells, which are immortalized Corti-derived epithelial cells, were provided by Professor F. Kalinec (UCLA, Los Angeles, CA, USA). The HEI-OC1 cell line was maintained in high-glucose Dulbecco’s modified medium (Life Technologies, Inc., NY, USA) supplemented with 10% fetal bovine serum (Life Technologies, Inc., NY, USA) and 0.06% w/v penicillin (Nacalai Tesque, Kyoto, Japan). The cells were incubated in 10% CO_2_ at 33 °C. This culturing medium also served as the control medium in the cell population and viability experiments. The maintenance of the HEI-OC1 cells in the above-mentioned condition is referred to as “permissive condition”.^23^ Every 3 to 4 days, the cell cultures were detached from the flasks using a 0.05 g/L trypsin and 0.53 mM EDTA solution (Nacalai Tesque, Kyoto, Japan), dissolved in Hank's buffered salt solution (HBSS) buffer, and subcultured in 10 ml growth medium per T-75 flask. The culture was maintained at 80–90% confluence.

### Cell viability assay and population doubling rate

The HEI-OC1 auditory cells (4 × 10^4^ cells/ml/well of 12-well plates) were incubated with multiple concentrations of H_2_O_2_ (0 μM, 100 μM, 300 μM or 1 mM; Nacalai Tesque, Kyoto, Japan), dissolved in the control medium listed in the cell culture and culture condition section for 1 h. The H_2_O_2_ solution was then replaced with culture medium lacking H_2_O_2_. The control cells were also prepared without H_2_O_2_. To determine viability, the cells were washed with Dulbecco’s phosphate-buffered saline (DPBS), which was harvested from the flasks via trypsinization (0.05w/v% trypsin, 0.53 mM EDTA, for 1 min), resuspended in DPBS, and diluted (1:1) in 0.4% trypan blue solution. The population doubling rate for HEI-OC1 cells was measured, as described previously.^24,50^ The cell population and viability were counted with a TC10 Automated Cell Counter (Bio-Rad, USA), following the manufacturer’s suggested procedures.

### Analysis of MMP and microscopic imaging

We analyzed disruption or loss of the MMP by JC-1 (Biotium, USA), which is capable of selectively entering the mitochondria to form JC-1 aggregates that emit red fluorescence at high MMP and form monomers that emit green fluorescence at low MMP.^51^ We also used tetramethylrhodamine, ethyl ester (TMRE) (Biotium, USA) to collect the images of morphological changes occurring in the mitochondria.

In MMP ratio assay with plate-reader analysis, the HEI-OC1 cells were cultured in ibiTreat micro-Plate 24-well (ibidi, Germany) and were exposed to H_2_O_2_ (0 μM, 100 μM, 300 μM or 1 mM) for 1 h and replaced with the normal medium on the day before the JC-1 analysis. The cells were incubated with JC-1 (200 nM; Biotium, USA) in fresh culture medium for 15 min at the permissive condition. Then, the cells were washed with culture medium and resuspended in fresh culture medium.

The fluorescence emission ratios were analyzed with Infinite M200 PRO plate-reader (TECAN, USA) with an incubator and CO_2_ controller. The cells were prepared on the plate, and the JC-1 emissions were collected at 535/20 nm with 485/9 nm excitation (green signal) and 590/20 nm with 535/9 nm excitation (red signal) under 10% CO_2_ incubation at 33 °C. The multiple reads per well (circle, 4 × 4) were performed, and the ratio of red/green was calculated.

In confocal microscopy, the HEI-OC1 cells were cultured in the glass-bottom dish (Greiner, Germany) and exposed to H_2_O_2_ under the same conditions as in the plate-reader analysis. The cellular mitochondria were stained with JC-1 (2.5 M), TMRE (200 nM) or MitoTracker Orange CMTMRos (500 nM) dye for 20 min and resuspended in fresh culture medium. The fluorescent images were collected using confocal microscope system (A1R, Nikon, Japan) with a 60 × (NA 1.4) oil immersion lens. JC-1 was excited with 487.5 nm or 561.4 nm semiconductor laser. TMRE was excited with 561.4 nm semiconductor laser. Fluorescence of JC-1 and TMRE were captured through a 405/488/561 nm dichroic mirror and 525/50 nm or 595/50 nm band-pass emission filter. The photomultipliers and pin-hole sizes were set to produce the clearest possible images without signal saturation. The imaging software NIS-Elements (Nikon, Japan) was used to process the raw recorded image data.

### Super-resolution live cell imaging using N-SIM

For structured illumination microscopic analyses, the cellular mitochondria were stained with TMRE dye and analyzed with a microscope (Ti-E; Nikon) equipped with a 100 × NA1.49 CFI Apo TIRF objective lens, an electron multiplying charged-coupled device camera (iXon Em-CCD, Andor), and imaging software NIS-Elements (Nikon, Japan).

### Image processing and analysis

Cells were dyed with MitoTracker Orange mitochondrial fluorescence, and then captured with confocal microscopy. All image processing and analysis were performed using NIS-Elements (Nikon, Japan) and ImageJ with Fiji, Skeletonize3D and AnalyzeSkeleton plugins.^52–54^ Focusing Regions were selected in mitochondria-rich parts of the cytoplasm. The red images are band-pass filtered image for mitochondrial shape, and the green lines are the filtered and skeletonized image for mitochondrial network (Fig. 4). Regarding mitochondrial network analysis, the objects were skeletonized using standard processing operations (medial axis transform), which involved an intensity threshold, followed by thinning and then pruning of the objects. Fine structural changes of mitochondria in cells were numerically analyzed. Parameters of mitochondrial morphology are described in Supplementary Table 1. The resulting mitochondrial skeleton was vectorized to identify and count/measure branches (skeletal backbone), end points, and branch points as graphic vectors/points (Fig. 4a). This was also used to measure distance map values (i.e. how far from the edge of the object any pixel/voxel lies) in order to determine branch diameter and volume. The natural logarithm ln(x) of raw parameter + 1 was used for statistical analysis because the parameter’s distribution was closed to log-normal distribution.

### TEM analysis

For TEM observation, the HEI-OC1 cells were harvested by trypsinization and fixed with 2% glutaraldehyde in 0.1 M sodium cacodylate buffer (pH 7.2) for 1 h, followed by 1% osmium tetroxide in 0.1 M sodium cacodylate buffer of the same pH for 2 h. The samples were en-bloc stained with 0.5% aqueous uranyl acetate overnight and dehydrated by alcohol at 4 °C and infiltrated with a graded series of Epon/Araldite mixture, followed by embedding in 100% Epon/Araldite. Thin sections (70 nM) were cut with a diamond knife, mounted on EM grids and stained with Reynolds’ lead citrate solution, followed by washing, drying, and imaging by using an H-7000 TEM (Hitachi, Tokyo, Japan).

### Measurement of oxygen consumption and extracellular acidification with XF24 Extracellular Flux Analyzer

The HEI-OC1 cells were seeded in the 24-well microplates of XF24 Extracellular Flux Analyzer (Seahorse Bioscience, Billerica, MA) with four blank background wells and incubated overnight. Then they were divided into four groups according to the Seahorse XF24 User’s Manual.

Then, the cells were treated without and with H_2_O_2_ (0 μM, 100 μM, 300 μM, 1 mM) for 1 h and incubated overnight. The cells were then rinsed twice in XF24 microplates and re-suspended in 675 μL of XF assay medium (DMEM without NaHCO_3_, 2 mM Glutamax; Seahorse Bioscience, Billerica, MA, USA), supplemented with 25 mM D-glucose (Otsuka Seiyaku, Tokushima, Japan) and 1 mM sodium pyruvate (Nacalai Tesque, Kyoto, Japan). After the cells were equilibrated for 1 h at 33 °C in a non-CO_2_ incubator prior to the assay, the oxygen consumption rate (OCR) was measured using the XF24 Extracellular Flux Analyzer where the cells were subjected to the following additions (in sequence): (1) basal levels were measured with no additives; (2) 2.5 μM oligomycin was added to inhibit ATP synthase and OXPHOS; (3) 1.25 μM FCCP to uncouple the proton gradient was added to induce maximal respiration; (4) 0.625 μM antimycin A was added to inhibit complex III; and (5) 0.625 μM rotenone was added to inhibit complex I, were added to end this reaction (Suppl. Fig. 1). Four separate measurements of OCR and extracellular acidification rate (ECAR) were taken after the addition of each inhibitor. Optimization of cell density and working concentration titers for each inhibitor was completed prior to the analysis according to the Seahorse XF24 User’s Manual (Suppl. Fig. 2). The OCR and ECAR were automatically calculated, recorded and plotted by Seahorse XF24 software (version 1.8.1). The all mitochondrial complex inhibitors were purchased as a kit from Seahorse Bioscience.

### mPTP analysis

We analyzed the mPTP with the TMRE fluorescence intensity decrement measurement under the FCCP triggered membrane potential depolarization. The HEI-OC1 cells were prepared in the same manner as in the confocal microscopy analysis, and the 200 μL fresh culture medium was resuspended. The TMRE fluorescence images of the cells were captured one at a time per minute under the same condition as the confocal microscopy imaging, and 1.375 μM or 2.75 μM FCCP was injected in a dosage of 50 μL per minute. The resulting final concentration is shown in Suppl. Fig. 3. The analyzed cells were selected as a region of interest (ROI) and average fluorescence intensity was plotted. The fluorescence intensities showed no difference during fluorescence image capture when FCCP was not injected.

For mPTP inhibition analysis, the HEI-OC1 cells were prepared in the same manner, and 1 μM CyA (Sigma; USA) was treated for 20 min before the analysis.

### Statistical analysis

Statistical analysis was computed using R version 3.2.4 software (R Core Team; R Foundation for Statistical Computing, Vienna, Austria, 2016), one-way analysis of variance, and Student’s t tests. *P*-values less than 0.05 were considered statistical significance. The data were expressed as means  ±  SD

## Electronic supplementary material

Supplementary Figure 1

Supplementary Figure 2

Supplementary Figure 3

Supplementary Figure 4

Supplementary Figure 5

Supplementary Table 1

## Electronic supplementary material

Supplementary Information accompanies the paper on the *npj Aging and Mechanisms of Disease* website (doi:10.1038/s41514-017-0002-2).
